# The Influence of pH and Temperature on the Stability of *N*-[(Piperidine)methylene]daunorubicin Hydrochloride and a Comparison of the Stability of Daunorubicin and Its Four New Amidine Derivatives in Aqueous Solutions

**DOI:** 10.1155/2014/803789

**Published:** 2014-02-06

**Authors:** Mikołaj Piekarski, Agnieszka Dołhań, Judyta Cielecka-Piontek, Przemysław Zalewski, Witold Kycler, Aleksandra Kaczmarek, Artur Firlej, Irena Oszczapowicz, Anna Jelińska

**Affiliations:** ^1^Department of Pharmaceutical Chemistry, Faculty of Pharmacy, Poznan University of Medical Sciences, 6 Grunwaldzka Street, 60-780 Poznań, Poland; ^2^Department of Oncological Surgery II, Greater Poland Cancer Centre, 15 Garbary Street, 61-688 Poznań, Poland; ^3^Department of Modified Antibiotics, Institute of Biotechnology and Antibiotics, 5 Starościńska Street, 02-515 Warsaw, Poland

## Abstract

The influence of pH and temperature on the stability of *N*-[(piperidine)methylene]daunorubicin hydrochloride (PPD) was investigated. Degradation was studied using an HPLC method. Specific acid-base catalysis of PPD involves hydrolysis of protonated molecules of PPD catalyzed by hydrogen ions and spontaneous hydrolysis under the influence of water zwitterions, unprotonated molecules, and monoanions of PPD. The thermodynamic parameters of these reactions, energy, enthalpy, and entropy, were calculated. Also, the stability of daunorubicin and its new amidine derivatives (piperidine, morpholine, pyrrolidine, and hexahydroazepin-1-yl) in aqueous solutions was compared and discussed.

## 1. Introduction

Anthracyclines are one of the most widely used groups of anticancer agents. The mechanism of action of anthracyclines, in addition to their multidirectional activity, involves direct intercalation of DNA, resulting in the biosynthesis of macromolecular stoppage, induction of oxidative stress inside cells by generating free radicals, combining with DNA and its alkylation, cross-linking of DNA strands, interrupting the DNA-helicase activity, direct impact on cell membranes and interruption of their activity, and induction of DNA damage and apoptosis with topoisomerase II activity stoppage. Each of these processes is closely connected with the structure of anthracyclines. Their application is limited by their dose-dependent cardiotoxicity, which was reported shortly after anthracyclines were introduced into clinical use. Another problem noted during anthracycline therapy is the growing drug resistance of cancer cells [1–4]. Anthracyclines are compounds of a glycoside structure, containing a sugar and an aglycone moiety. The sugar is usually daunosamine whereas the aglycone moiety consists of four six-carbon rings. The complex structure of anthracyclines allows many modifications of both the aglycone and the sugar moiety [5–10]. Many novel anthracycline derivatives modified in the sugar moiety were synthesized in the Institute of Biotechnology and Antibiotics, Warsaw, Poland [11]. It was proved that a group of 3′-formamide-substituted daunorubicin derivatives shows a much lower tendency to produce free radicals. Consequently, their antitumor activity, similar to that of daunorubicin, is combined with lower toxicity to the healthy cells of the body. Derivatives modified in the daunosamine moiety, in addition to anticancer activity, are able to break the resistance barrier. As those compounds do not target topoisomerase II, the cells containing the mutated enzyme are still sensitive to those compounds [12].

**Figure 1:**
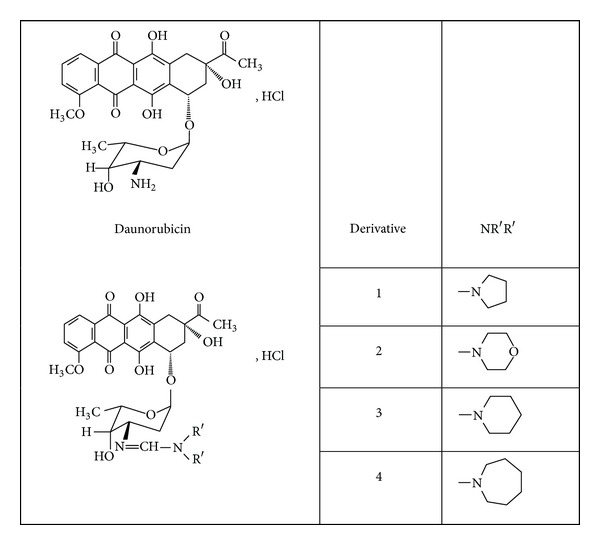
The chemical structure of daunorubicin and its four amidine derivatives.

The new derivatives of daunorubicin containing in the amidine group a piperidine, morpholine, pyrrolidine, or hexahydroazepine moiety (Figure 1) demonstrate antiproliferative activity similar to daunorubicin [5, 13, 14]. It was found that daunorubicin was degraded in aqueous solutions and in the solid state [15, 16]. Its degradation in aqueous solution was studied at 50°C and pH 0–14. The photodegradation of daunorubicin [17] and stability of daunorubicin in infusion fluids have also been studied [18–23]. Daunorubicin stored in polypropylene syringes at 4°C after diluting with water for injections was stable at least 43 days [18]. Studies of the stability and compatibility of a mixture of etoposide, cytarabine, and daunorubicin have shown that all of them were stable in 5% glucose solution, both when separate and mixed. The greatest stability was obtained by storing the mixture in the dark at room temperature [19].

The aim of our work was to evaluate the stability of *N*-[(piperidine)methylene]daunorubicin hydrochloride (PPD) in a wide range of pH and to determine the pH range in which it is the most stable. Also, the stability of daunorubicin and its four amidine derivatives was compared.

In order to establish the observed rate constants an isocratic HPLC method was used.

## 2. Materials and Methods

### 2.1. Samples

*N*-[(Piperidine)methylene]daunorubicin hydrochloride (PPD) was synthesized in the Department of Modified Antibiotics, Institute of Biotechnology, Warsaw, Poland [11]. PPD is a reddish powder, freely soluble in water and methanol. Quinine hydrochloride (Sigma-Aldrich Logistik GmbH, Germany) was used as an internal standard. Sodium laurisulfate (A.C. reagent Sigma-Aldrich Logistik GmbH, Germany) and all other chemicals and solvents were obtained from Merck KGaA, Germany, and were of analytical or high-performance liquid chromatographic grade.

### 2.2. Chromatographic Conditions

Chromatographic separation and quantitative analysis were performed by using an HPLC method [24]. The analytical system consisted of a Shimadzu SPD-20A Prominence UV/VIS detector and a Rheodyne with a 50 *μ*L loop. An LiChrospher RP-18 column (125 mm × 4 mm, 5 *μ*m particle size, Merck, Germany) was used as the stationary phase. The mobile phase consisted of a mixture of 9 volumes of acetonitrile, 1 volume of methanol, and 10 volumes of a solution containing 2.88 g L^−1^ of sodium laurisulfate and 1.6 mL L^−1^ of phosphoric acid. The flow rate was 1.5 mL min^−1^ and UV detection was performed at 254 nm. Although the method was evaluated and validated for the determination of four derivatives of daunorubicin, the selectivity was examined during a stability study of PPD.

### 2.3. Kinetic Procedures

The degradation of PPD in aqueous solutions was studied at 313, 325, 333, and 343 K in the pH range 0.43–5, at 303, 308, and 313 K at pH above 5.5, and in sodium hydroxide solutions at 298, 303, 308, and 313 K. The pH values of the reaction solutions and those of the buffer standards were measured at reaction temperatures. The pH values of the reaction solutions in HCl and NaOH were calculated from the equations pH = −log⁡*f*_HCl_[HCl] or pH = *pK*_*w*_ + log⁡*f*_NaOH_[NaOH]. The activity coefficients *f*_HCl_ and *f*_NaOH_ were obtained or calculated from the literature data [25]. The ionic strength of all the solutions was adjusted to 0.50 mol L^−1^ with a solution of sodium chloride (4 mol L^−1^). Solutions of the desired pH and ionic strength of 0.50 mol L^−1^ were heated to the required temperatures and then a sample of PPD was added. The initial concentration of PPD was 0.2 mg mL^−1^. At selected times, determined by the rate of degradation, samples of the solutions (0.5 mL) were collected and instantly cooled with a mixture of ice and water. Samples with pH above 7.5 were neutralized by using HCl solutions at concentrations ensuring that their pH was approximately 2. To such samples, 0.5 mL of the internal standard solution (quinine hydrochloride solution) was added. 50 *μ*L of samples of the solutions was injected in the column.

## 3. Results and Discussion

### 3.1. Observed Rate Constants

The observed rate constants of PPD degradation were determined in a pH range of 0.43–13.54 and were described by the equation of a pseudo-first-order reaction:

(1) $$\begin{array}{c} \ln c_{t}=\ln c_{0}-k_{\text{obs}}\times t, \end{array}$$

where *c*_*t*_ and *c*_0_ are the time-dependent concentration and the initial concentration of PPD at time *t* > 0 and *t* = 0, respectively, *k*_obs_ is the observed rate constant of the pseudo-first-order reaction of PPD degradation. The number of measurements of *c*_*t*_ for each series ranged from 8 to 12.

### 3.2. Buffer Catalysis

Under the conditions of this study, the rate constants (*k*_obs_) did not depend on the total concentrations of the phosphate, acetate, and borate buffers, which indicated that the components of the buffers did not catalyze the degradation of PPD. To verify that the differences between *k*_obs_ determined at different buffer concentrations were not statistically significant, the parallelism test was used. Since in the reaction solutions of PPD in HCl, phosphate, acetate, and borate buffers as well as in NaOH, general acid-base catalysis was not observed, in the whole range of pH the values of *k*_obs_ = *k*_pH_.

### 3.3. pH-Rate Profiles

**Figure 2:**
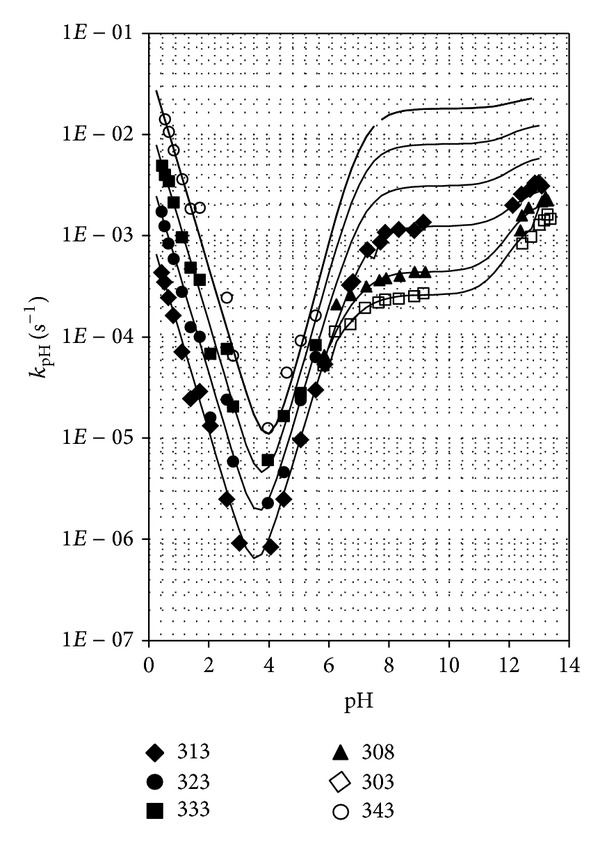
log *k*_pH_ = *f*(pH) profiles for the degradation of PPD in aqueous solutions. The points are determined experimentally and the lines were calculated from (2).

The rate constants *k*_pH_ determined in hydrochloric acid, sodium hydroxide, phosphate, borate, and acetate buffers were used to calculate the relationship log *k*_pH_ = *f*(pH) (Figure 2).

The semilogarithmic relationship *k*_pH_ − pH indicated that in water solutions at pH 0.43–13.54, the following reactions occurred.

- Degradation of protonated molecules of PPD catalyzed by hydrogen ions (*k*_1_),
- Spontaneous hydrolysis of zwitterions (*k*_2_), unprotonated molecules (*k*_3_), and monoanions (*k*_4_) of PPD under the influence of water.

The total reaction rate was equal to the sum of partial reaction rates:

(2) $$\begin{array}{c} k_{\text{pH}}=k_{1}a_{{\text{H}}^{+}}f_{1}+k_{2}f_{2}+k_{3}f_{3}+k_{4}f_{4}, \end{array}$$

where *a*_H^+^_ is the hydrogen ion activity and *f*_1_–*f*_4_ are the fractions of the molecules of PPD.

**Figure 3:**
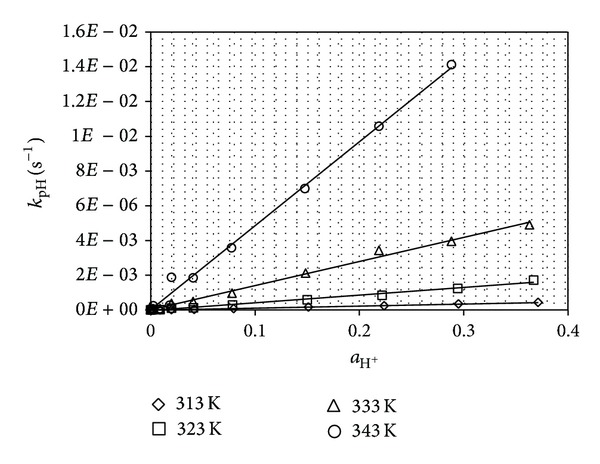
Plots *k*_pH_ = *f*(*a*_H^+^_) for the degradation of PPD in aqueous solutions.

**Figure 4:**
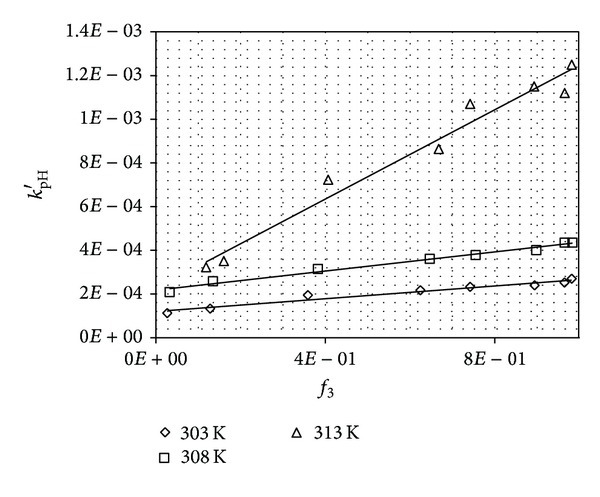
Plots *k*_pH_′ = *f*(*f*_3_) for the degradation of PPD in aqueous solutions.

The catalytic rate constants *k*_1_ were calculated from the plots *k*_pH_ = *f*(*a*_H^+^_), which were linear with a positive slope that was equal to *k*_1_ (Figure 3). The catalytic rate constants of the spontaneous hydrolysis of PPD monoanions were calculated as the mean values of *k*_pH_ at pH above 12. The catalytic rate constants *k*_2_ and *k*_3_ were calculated from the equation *k*_pH_′ = *f*(*f*_3_) using the *k*_pH_ values at pH 5.5–9 where the concentrations of forms *f*_2_ + *f*_3_ → 1 (*k*_pH_′ = *k*_pH_(*k*_1_ *a*_H^+^_ *f*_1_ + *k*_4_ *f*_4_)) (Figure 4). The plots *k*_pH_′ = *f*(*f*_3_) were linear and the value *k*_pH_′ for *f*_3_ = 1 was equal to the catalytic rate constant *k*_3_, whereas the value of *k*_pH_′ for *f*_3_ = 0 corresponded to the catalytic rate constant *k*_2_.

The values of *k*_2_, *k*_3_, and *k*_4_ were calculated at 303, 308, and 313 K and next extrapolated from the Arrhenius relationship to 323, 333, and 343 K. The calculated theoretical profile of log⁡⁡*k* = *f*(pH) and that obtained from the experimental results were nearly identical, indicating that the choice of the equation describing the total rate of PPD degradation was correct (Figure 2).

### 3.4. Influence of Temperature

Based on the Arrhenius relationship ln *k* = ln⁡*A* − *E*_*a*_/*RT*, linear plots of ln *k* = *f*(1/*T*) were used to calculate the energy of activation (*E*_*a*_), the entropy (Δ*S* ^≠^) and enthalpy (Δ*H* ^≠^), and the preexponential coefficient (*A*) for the partial reactions (Table 1). The lowest energy of activation was observed in the reaction of spontaneous hydrolysis of PPD zwitter ions. The entropy of all reactions under the influence of water (spontaneous hydrolysis) was negative, which suggested the bimolecular character of these reactions. The positive values of entropy for the reactions of protonated molecules of PPD catalyzed by hydrogen ions indicated a positive participation of entropy of protonation reaction.

**Figure 5:**
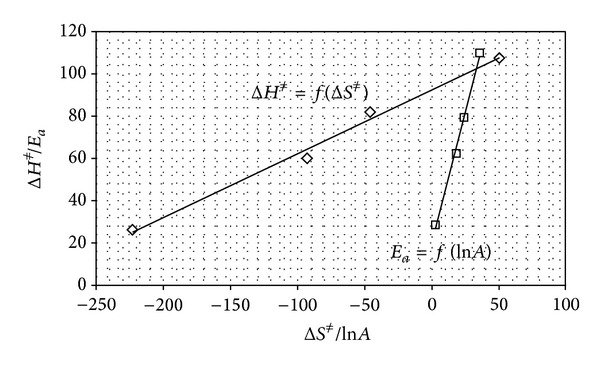
The relationships Δ*H* ^≠^ = *f*(Δ*H* ^≠^) and *E*_*a*_ = *f*(ln⁡⁡*A*) for the hydrolysis of PPD^+^ catalyzed by hydrogen ions and spontaneous hydrolysis of PPD^±^, PPD, and PPD^−^ under the influence of water.

The linear relationships of Δ*H* ^≠^ = *f*(Δ*H* ^≠^) and *E*_*a*_ = *f*(ln⁡*A*) (Figure 5) were obtained for the degradation of protonated molecules of PPD catalyzed by hydrogen ions and spontaneous hydrolysis of PPD^±^, PPD, and PPD^−^ molecules under the influence of water, which suggested that all reactions occurred according to the same mechanism of a bimolecular reaction.

### 3.5. Influence of Ionic Strength

The influence of ionic strength was studied in hydrochloric acid (0.10 mol L^−1^, 343 K) and sodium hydroxide solution (0.10 mol L^−1^, 308 K) and interpreted according to the Brönsted-Bjerrum equation. It was found that in the solutions of hydrochloric acid a positive salt effect was observed (the slope (*a*) of the relationship log *k*_obs_ = *f*(√*μ*/(1 + √*μ*)) was 1.29 ± 0.63, whereas in the sodium hydroxide solutions no influence of the ionic strength was observed (*a* = 0.051). These results confirmed that in hydrochloric acid the degradation of PPD occurred as a reaction of protonated molecules of PPD catalyzed by hydrogen ions and in the sodium hydroxide solutions as a spontaneous hydrolysis of PPD under the influence of water.

## 4. A Comparison of the Stability of Daunorubicin and Its Four New Amidine Derivatives in Aqueous Solutions

Specific acid-base catalysis of daunorubicin involves reactions catalyzed by hydrogen and hydroxyl ions and spontaneous hydrolysis under the influence of water depending on the DAU charge [15] whereas specific acid-base catalysis of the new amidine derivatives of DAU comprises hydrolysis of protonated molecules and spontaneous hydrolysis under the influence of water depending on the charge of the derivatives [26–28]. In the case of amidine, DAU derivatives hydrolysis catalyzed by hydroxide ions is not observed.

**Figure 6:**
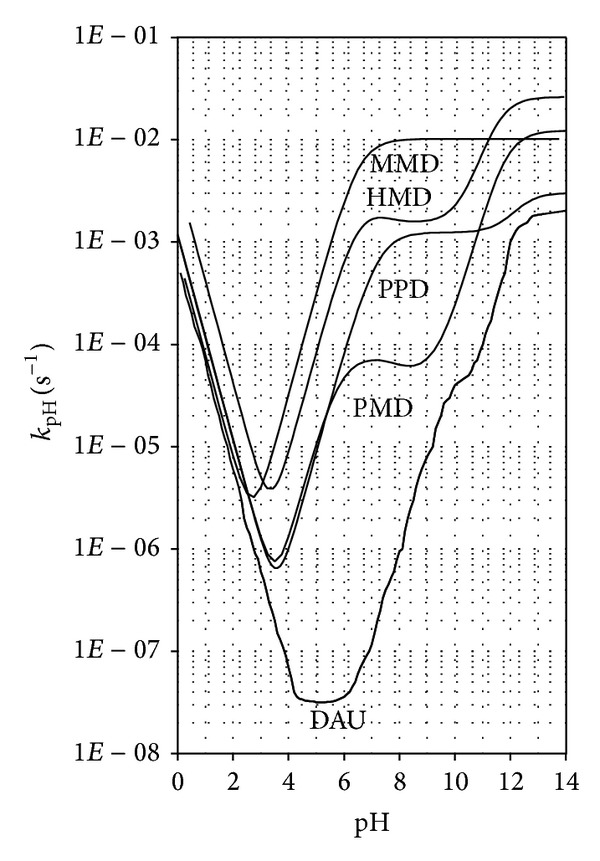
log *k*_pH_ = *f*(pH) profiles of daunorubicin (DAU) and its four amidine derivatives: *N*-[(pyrrolidine)methylene]daunorubicin (PMD), *N*-[(morpholine) methylene]daunorubicin (MMD), *N*-[(piperidine) methylene]daunorubicin (PPD), and *N*-[(hexahydroazepin-1yl) methylene]daunorubicin (HMD) in aqueous solutions at 313 K.

By comparing the relationship log *k*_pH_ = *f*(pH) for daunorubicin (DAU) and for its four amidine derivatives, pyrrolidine (PMD), morpholine (MMD), piperidine (PPD), and hexahydroazepin-1-yl (HMD) [15, 26–28] (Figure 6), it is possible to conclude the following:

- daunorubicin demonstrates the greatest stability in the pH range 4–6;
- in the entire pH range daunorubicin is the most stable compound;
- the smallest differences in the rate of degradation of DAU and its four derivatives are observed in an acidic environment, where the dominant reaction is the degradation of protonated molecules catalyzed by hydrogen ions;
- significant differences in the degradation rate are observed in spontaneous hydrolysis under the influence of water;
- in the pH range 4–6, in which DAU demonstrates the greatest stability, its derivatives undergo degradation at a greater rate from 3 (PMD) to 5 orders (MMD) of magnitude.
- MMD demonstrates the greatest stability at pH about 2.5 whereas PPD, PMD, and HMD at about 3.5;
- in the pH range 6–10 the stability of DAU and its four derivatives compares as follows: DAU ≫ PMD > PPD > HMD > MMD.

## Figures and Tables

**Table 1:** Kinetic and thermodynamic parameters for the degradation of PPD in aqueous solutions.

*T* (K)	*k* ± Δ*k* (s^−1^)	Statistical evaluation ln *k*_*i*_ = *f*(1/*T*)	Thermodynamic parameters
313	(1.17 ± 0.05) · 10^−3^	*r* = − 0.999 ** ** *a* = − 13220 ± 1010 ** ** *b* = 35.5 ± 3.1	*E*_*a*_ = 109.9 ± 8.4 (kJ·mol^−1^) Δ*H* ^≠^ = 107.5 ± 8.4 (kJ·mol^−1^) Δ*S* ^≠^ = 50.2 ± 25.6 (J·K^−1^·mol^−1^)
323	(4.40 ± 0.03) · 10^−3^		
333	(1.39 ± 0.08) · 10^−2^		
343	(4.84 ± 0.03) · 10^−2^		

303	1.19 · 10^−4^	*r* = − 0.850 *a* = − 3440 ± 9162 *b* = 2.64 ± 30.1	*E*_*a*_ = 28.6 ± 76.2 (kJ·mol^−1^) Δ*H* ^≠^ = 26.2 ± 76.2 (kJ·mol^−1^) Δ*S* ^≠^ = − 222.8 ± 250.2 (J·K^−1^·mol^−1^)
308	2.01 · 10^−4^		
313	2.21 · 10^−4^		

303	2.66 · 10^−4^	*r* = − 0.999 *a* = − 9555 ± 1065 *b* = 23.8 ± 3.5	*E*_*a*_ = 79.4 ± 8.9 (kJ·mol^−1^) Δ*H* ^≠^ = 81.9 ± 8.9 (kJ·mol^−1^) Δ*S* ^≠^ = − 45.9 ± 29.0 (J·K^−1^·mol^−1^)
308	4.36 · 10^−4^		
313	1.23 · 10^−3^		

303	0.970 · 10^−3^	*r* = − 0.999 *a* = − 7509 ± 690 *b* = 18.3 ± 2.3	*E*_*a*_ = 62.4 ± 5.7 (kJ·mol^−1^) Δ*H* ^≠^ = 60.0 ± 5.7 (kJ·mol^−1^) Δ*S* ^≠^ = − 92.9 ± 18.8 (J·K^−1^·mol^−1^)
308	1.50 · 10^−3^		
313	2.26 · 10^−3^		
318	3.23 · 10^−3^		

Δ*H* ^≠^and Δ*S* ^≠^were calculated for 298 K. *E*_*a*_ = − *aR* (J mol^−1^); Δ*H* ^≠^ = *E*_*a*_ − RT (J mol^−1^); Δ*S* ^≠^ = *R*  (ln⁡*A* − ln⁡ (*k*_*B*_ *T*)/*h* (J K^−1^ mol^−1^), where *k*_*B*_: Boltzmann constant (1.3807 10^−23^ J K^−1^); *h* = Planck constant (6.626 10^−34^ J s); *R* = universal gas constant (8.314 J K^−1^ mol^−1^); *T*: temperature in K; *a*: vectorial coefficient of the Arrhenius relationship; *A*: frequency coefficient.
